# Detection of Anemia in Schoolchildren Aged 6–18 Years With Hematocrit Percentile Charts and the Impact of Economic Status in Southern Brazil

**DOI:** 10.1002/ajhb.70034

**Published:** 2025-03-31

**Authors:** Vanessa Regina Jung, Nikolas Mateus Pereira de Souza, Dhuli Kimberli Abeg da Rosa, João Francisco de Castro Silveira, Cézane Priscila Reuter, Alexandre Rieger

**Affiliations:** ^1^ Postgraduate Program in Health Promotion University of Santa Cruz Do Sul Santa Cruz do Sul Rio Grande do Sul Brazil; ^2^ Department of Life Sciences University of Santa Cruz Do Sul Santa Cruz do Sul Rio Grande do Sul Brazil; ^3^ Bioprocess Engineering and Biotechnology State University of Rio Grande Do Sul Santa Cruz do Sul Rio Grande do Sul Brazil; ^4^ Postgraduate Program in Environmental Technology University of Santa Cruz Do Sul Santa Cruz do Sul Rio Grande do Sul Brazil

**Keywords:** anemia, hematocrit, LMS method, percentile charts

## Abstract

**Objective:**

To generate hematocrit percentile charts for schoolchildren aged 6–18 years and determine the prevalence of anemia by socioeconomic status class in southern Brazil.

**Methods:**

This is a cross‐sectional study utilizing data collected between 2014 and 2017 from southern Brazil. The study's sample consists of 4802 schoolchildren, aged 6 to 18 years. The percentile charts for sex‐specific hematocrit were developed using the LMS (Lambda‐Mu‐Sigma) method. The simplified economic classification, based on ABEP criteria, was used to group individuals into A + B (high), C (middle), and D + E (low) income classes. Anemia was defined as hematocrit z‐score ≤ −1.96 for age and sex.

**Results:**

Among boys, 58 (2.86%) were anemic, 1955 (94.81%) had normal hematocrit levels, and 48 (2.33%) had high hematocrit. Girls showed a similar pattern, with 73 (2.73%) anemic, 2534 (94.90%) with normal hematocrit, and 63 (2.36%) with high hematocrit. For girls, a higher prevalence of non‐anemic hematocrit was observed in class A (39.33%) compared to anemic children (23.28%), with significant standardized residuals. For boys, significant residuals were observed for a higher prevalence of anemic children in the lower socioeconomic class DE (13.79%) compared to non‐anemic children (5.18%), and a higher prevalence of non‐anemic children in the upper socioeconomic class A (42.63%) compared to anemic children (22.41%).

**Conclusion:**

The percentile charts generated from hematocrit levels enabled the comparison of anemia prevalence across socioeconomic status classes. A higher prevalence of anemia was found among boys in lower socioeconomic classes, while girls in higher socioeconomic classes showed a lower prevalence of anemia.

## 1 Introduction

Anemia is a widespread public health issue, particularly affecting vulnerable populations such as young children, pregnant and postpartum women, and menstruating adolescent girls (Brittenham et al. 2023). Defined as a condition where the number of red blood cells or hemoglobin concentration is below normal, anemia is diagnosed based on blood hemoglobin levels falling below specific thresholds established for age, sex, and physiological status (World Health Organization 2025). Hemoglobin, a protein in red blood cells, is essential for transporting oxygen throughout the body, while hematocrit refers to the proportion of blood volume occupied by red blood cells (Brittenham et al. 2023). Anemia manifests with symptoms like fatigue, shortness of breath, and reduced physical capacity. Iron deficiency is the leading cause of anemia worldwide, accounting for over 60% of cases, particularly in low‐ and lower‐middle‐income settings (World Health Organization 2025). Other significant causes include genetic disorders, chronic and acute blood loss, inadequate nutritional intake, infectious diseases like malaria, and deficiencies in key nutrients such as vitamin A, folate, vitamin B12, and riboflavin (Shekar et al. 2017; Wiafe et al. 2023). These factors, individually or in combination, impair hemoglobin synthesis and red blood cell production, compounding the prevalence of anemia.

The impact of anemia extends beyond physical health, especially in children. In school‐age populations, it poses serious risks to cognitive and physical development, often resulting in developmental delays, behavioral disturbances, and decreased motor activity (Pivina et al. 2019). This condition can diminish attention spans and social interaction, directly affecting academic performance and future productivity (Gutema et al. 2023). Anemia also serves as a critical indicator of poor nutrition and overall health, particularly in regions where nutrient deficiencies, parasitic infections, and inadequate healthcare infrastructure are prevalent (Shekar et al. 2017). Addressing anemia requires a comprehensive approach, integrating dietary improvements, disease prevention, and public health interventions to mitigate its far‐reaching consequences on quality of life and economic development.

Puberty is also associated with significant changes in the activity of sex hormones, which directly influence erythropoiesis by stimulating the production of erythropoietin, a key regulator of red blood cell synthesis (Bachman et al. 2014; Mancera‐Soto et al. 2021). In boys, increased testosterone levels during this phase enhance red blood cell production and oxygen transport capacity, supporting the elevated metabolic demands associated with greater muscle mass (Mairbäurl 2013). In contrast, the influence of estrogen on erythropoiesis in girls is less pronounced, and factors such as blood loss due to menstruation can contribute to slightly lower hematocrit levels. These physiological differences result in sex‐specific variations in hematological parameters during puberty. Percentile charts and Z‐scores account for these continuous changes associated with aging, enabling accurate assessment of the gradual transition between test results considered normal and those potentially classified as pathological (Zierk et al. 2019).

Differences in hematological variables between sexes begin to emerge after the onset of menstruation and persist until approximately 10 years after menopause (Murphy 2014). Menstruation and nutritional intake are the main reasons for lower hemoglobin and iron levels in women compared to men (Murphy 2014). During puberty, the total amount of hemoglobin tends to increase more in boys than in girls (Gligoroska et al. 2019). Increases in hematocrit levels in boys during adolescence are likely more related to sexual maturation than chronological age, as androgen secretion is responsible for these changes (Mancera‐Soto et al. 2021).

The numerous underlying causes of anemia significantly heighten the vulnerability of adolescents, a group whose bodies are in a critical stage of rapid growth, demanding an elevated intake of essential micronutrients to support optimal development (Gore et al. 2024). This age group experiences an intensified physiological need for nutrients like iron, especially during puberty, when body mass and tissue growth require increased oxygen transport and energy production. However, studies have shown that many adolescents struggle to meet these nutritional demands, largely due to a diet low in foods rich in bioavailable iron, such as lean meats, fish, and leafy green vegetables (Pasricha et al. 2013). Additionally, adolescents often face a diet skewed towards highly processed foods with low nutrient density, which compounds their risk for anemia. The prevalence of anemia in this demographic is not only a reflection of inadequate iron intake but also of increased iron demands as the body works to expand tissues, increase red blood cell mass, and support the development of major organs (Gore et al. 2024).

In Brazil, the short duration of exclusive breastfeeding, diarrhea, and lack of basic sanitation have been identified as significant contributors to low hemoglobin levels among children and adolescents (Leal and Osório 2010; Oliveira et al. 2011; Zanin et al. 2015). These factors are often intertwined with broader socioeconomic inequalities that exacerbate health disparities. A substantial body of evidence supports the notion that anemia serves as a marker of socioeconomic disadvantage (Cardoso et al. 2012; Oliveira et al. 2011; Zanin et al. 2015). Children and adolescents from the poorest households and with less educated caregivers are at the highest risk of exposure to conditions that contribute to anemia and its associated complications.

A study conducted by (Cardoso et al. 2012), with children aged 6 months to 10 years in northwestern Brazil, found that socioeconomic factors were significant predictors of anemia. Specifically, the risk of anemia among children living in households within the lowest wealth quartile was 40% higher compared to those in the highest wealth quartile. This finding underscores the critical role that economic conditions play in determining health outcomes, as financial constraints often limit access to nutritious foods, healthcare services, and adequate living conditions—all of which are essential for preventing anemia.

Given the importance of population‐specific percentile curves for schoolchildren, our objective was to generate sex‐specific hematocrit percentile curves in a city in southern Brazil to facilitate early anemia screening. Furthermore, we aimed to associate the different prevalences of anemia according to socioeconomic status.

## 2 Methods

### 2.1 Participants

The study involved schoolchildren who participated in two phases of the “Schoolchildren's Health” research initiative, a cross‐sectional study utilizing data from schoolchildren who participated in phases III (2014–2015) and IV (2016–2017) by the University of Santa Cruz do Sul (UNISC) in Santa Cruz do Sul, RS, Brazil. This initiative, consisting of school‐based cross‐sectional studies, began in 2004 and has been conducted periodically, aimed at assessing various health‐related outcomes among schoolchildren within the municipality. Data collection took place from March to December, covering all seasons except summer.

The sample comprised 4802 students aged 6 to 18 years, enrolled in both public and private schools. Schools and students were randomly selected through cluster sampling, ensuring representation aligned with the population density of the municipality's regions (north, south, east, west, and central), including both urban and rural areas. A total of 25 schools participated in the evaluations during both research phases analyzed in this study. Within the selected schools, specific classes were chosen, and all students within these classes were invited to participate. Students were included if they were aged 6 to 18 years, and those incapable of performing physical tests, completing questionnaires, or providing biological samples (e.g., blood) due to contraindications were excluded. In instances where subjects were analyzed in subsequent years, only data from the first interview conducted during the initial year of data collection were included in the analysis. Consequently, no replicated subjects are present.

The study received ethical approval from the UNISC ethics committee (CAEE: 31576714.6.0000.5343 and CAEE: 54982616.7.0000.5343). For students under 18 years old, participation required parental consent through the signing of the Free and Informed Consent Form, while 18‐year‐old students provided their own consent by reading and signing the form. Additionally, the school enrollment records of each participant were consulted to classify their residence location as rural or urban, based on the territorial map delineating the distribution between rural and urban areas by municipality, as provided by the Brazilian Institute of Geography and Statistics (IBGE) on its official website (IBGE 2024).

### 2.2 Classification of Socioeconomic Status

The simplified classification of economic status was generated according to the criteria proposed by the Brazilian Association of Research Companies (ABEP), with a year‐specific questionnaire for each year of data collection. The simplified classification consists of six family income classes: A, B1, B2, C1, C2, and DE. For statistical purposes, these classes were grouped as follows: A + B (B1 + B2), C (C1 + C2), and D + E. Class A + B is considered to be families with high income, C with medium income, and D + E with low income. The ABEP points system is determined based on variables including the number of bathrooms in the household, ownership of cars and motorcycles, the types and quantities of household appliances, the educational attainment of the household head, and access to public services. Table S1 in the Supporting Information provides a summary of the variation in average household income for each socioeconomic class defined by ABEP between 2014 and 2017.

### 2.3 Blood Collection and Analysis

Blood collection for the evaluation of hematological parameters was collected by a trained professional on a prescheduled day. Blood was drawn from the antecubital vein in 4 mL K_2_EDTA tubes (VacuetteVR, Greiner Bio‐One). Samples were collected in the university, and the children had not participated in strenuous exercise prior to sample collection. At the end of each collection session, samples were transported to the laboratory of the university. The hematology analyses were performed on a Sysmex XS‐800i analyzer with reagents from the supplier. Only hematocrit was used for the present study.

### 2.4 Construction of Percentile Charts

The percentile charts for sex‐specific hematocrit were developed using the LMS (Lambda‐Mu‐Sigma) method (Cole and Green 1992). This method describes the distribution of data across ages by three curves: median (M, μ), coefficient of variation (S, σ), and skewness (L, λ), the latter expressed as a Box‐Cox power. These curves are fitted by non‐linear regression with cubic splines, using penalized likelihood. The optimization and smoothing of the curves is determined by the equivalent degrees of freedom (edf). A grid search was performed with a loop varying the values of edf, starting with L (edf) = 0, M (edf) = 1, and S (edf) = 1 until reaching L (edf) = 3, M (edf) = 7, and S (edf) = 7, generating 147 models with the combination of parameters. The model that obtained the lowest Bayesian Information Criterion (BIC) deviance measure value was chosen. The curves were generated with 7 centiles: 3rd, 10th, 25th, 50th, 75th, 90th, and 97th.

The curve of the velocity of hematocrit variation per year is obtained with the 1st derivative of the M curve. The z‐scores are extracted from the L, M, and S curves when L > 1, and obtained from the conventional equation when L = 1 (normal distribution). The percentile charts, velocity, and z‐score were determined with the LMSchartmaker Pro software (version 2.54, Medical Research Council, UK).

### 2.5 Statistical Analysis

The schoolchildren were categorized by sex using frequency analysis, considering factors such as race (white, black, mixed, indigenous, and Asian), area of residence (urban or rural), pubertal stage (prepubescent, pubescent, and postpubescent), and economic status. Pubertal stages were classified through self‐assessment using the Tanner stage scale, which is specific for boys and girls. For statistical analysis, simplified Tanner stages were used: prepubescent (stage I), pubescent (stages II, III, and IV), and postpubescent (stage V).

Three hematocrit classes were defined by stratifying the z‐scores extracted from the percentile charts (in standard deviations, SD) as follows: Class A (high hematocrit): ≥ 1.96; Class B (normal hematocrit): −1.96 to 1.96; Class C (anemia): ≤ −1.96. These classes were used to determine the prevalence of elevated hematocrit, normal hematocrit, and anemia in our sample. For the association analysis using the chi‐square test, classes A and B were combined, aiming to analyze the association of economic status directly with anemia. For significance analysis in the multiclass chi‐square test, standardized residuals were used. A *p*‐value < 0.05 was considered significant, and a standardized residual > |1.96| was considered significant.

## 3 Results

### 3.1 Sample Description

The description of the sample by sex is presented in Table 1. A homogeneous proportional distribution of race and socioeconomic status between the sexes was observed. However, there was a difference in the proportion of students from urban and rural areas between the sexes, with 81.90% of boys from urban areas and 18.10% from rural areas, compared to 85.16% of girls from urban areas and 14.84% from rural areas. The greatest proportional difference was observed in the distribution between pubertal stages, with 27.34% of girls in the pubertal stage compared to 17.99% of boys. Conversely, there were more boys in the prepubertal (48.16%) and postpubertal (33.85%) stages than girls in the prepubertal (44.87%) and postpubertal (27.79%) stages, respectively. Standardized residuals were > |1.96| in all multiple comparisons of zone and pubertal status.

**TABLE 1:** Description of the study sample of schoolchildren aged 6 to 18 years, stratified by sex.

	Boys	Girls	Total	*p*
Race				
White	1595 (77.62%)	2044 (76.93%)	3639 (77.23%)	0.723
Black	169 (8.22%)	206 (7.75%)	375 (7.96%)	
Mixed	262 (12.75%)	373 (14%)	635 (13.48%)	
Indigenous	10 (0.49%)	13 (0.49%)	23 (0.49%)	
Asian	19 (0.93%)	21 (0.80%)	40 (0.85%)	
Zone				
Urban	1688 (81.90%)	2273 (85.16%)	3961 (83.74%)	0.003
Rural	373 (18.10%)	396 (14.84%)	769 (16.26%)	
Pubertal status				
Prepubertal	993 (48.16%)	1198 (44.87%)	2191 (46.30%)	< 0.001
Pubertal	371 (17.99%)	730 (27.34%)	1101 (23.27%)	
Postpubertal	698 (33.85%)	742 (27.79%)	1440 (30.43%)	
Socioeconomic status				
A + B	861 (42.06%)	1031 (38.89%)	1892 (40.27%)	0.061
C	1075 (52.52%)	1452 (54.77%)	2527 (53.79%)	
D + E	111 (5.42%)	168 (6.34%)	279 (5.94%)	

*Note:* Socioeconomic status classes according to the Brazilian Association of Research Companies (ABEP), being A + B (high), C (middle), and D + E (low) income classes. Simplified Tanner stages were used: prepubescent (stage I), pubescent (stages II, III, and IV), and postpubescent (stage V). *p*‐value derived from chi‐square test.

### 3.2 Comparison of Percentile Charts for Boys and Girls and Prevalence of Anemia

**FIGURE 1:**
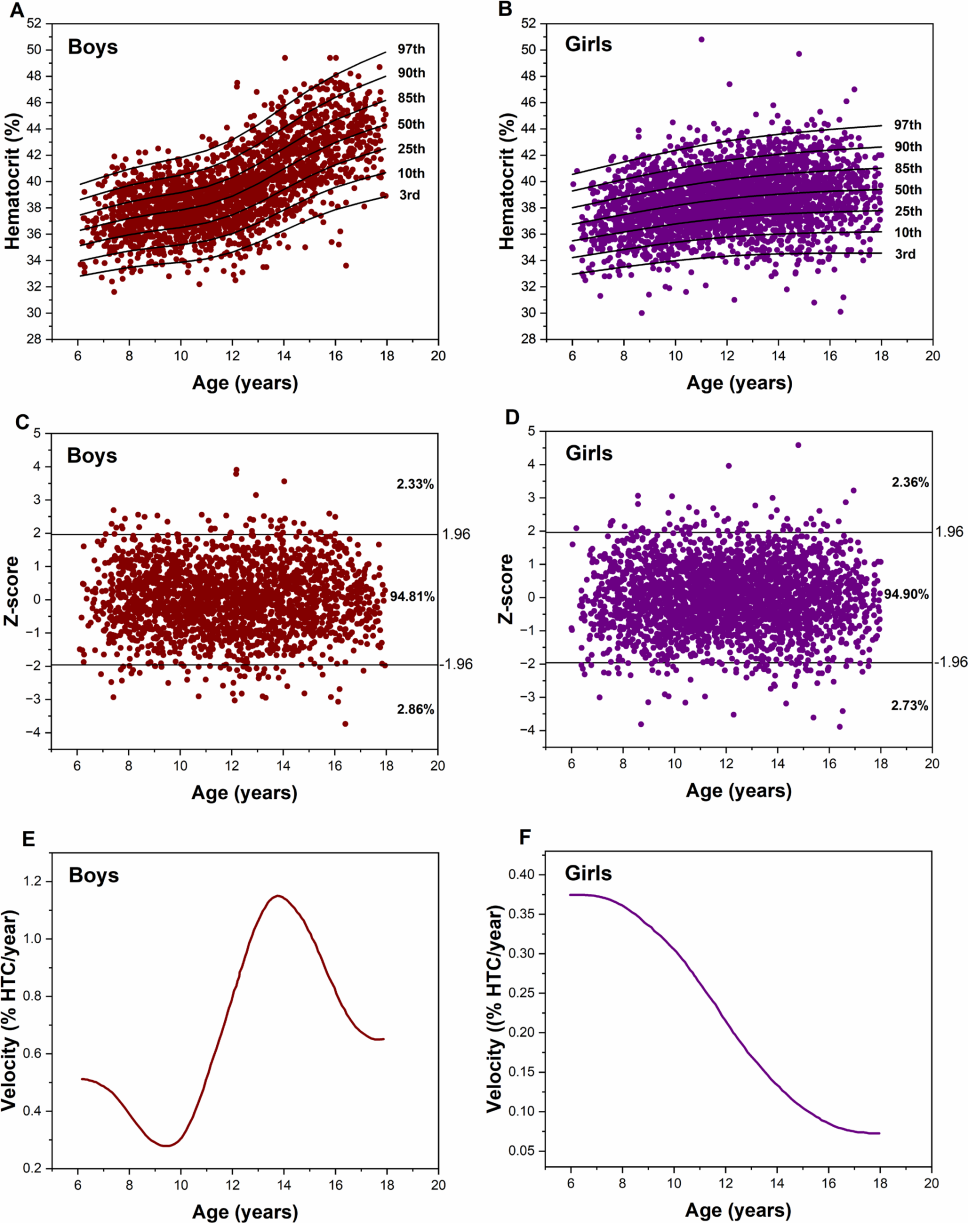
Percentile charts, z‐scores, and rate of change of hematocrit for schoolchildren aged 6–18 years. (A) Percentile chart with 7 percentiles for boys. (B) Percentile chart with 7 percentiles for girls. (C) Z‐scores derived from the boys' percentile chart with two horizontal lines (−1.96 and 1.96) indicating the prevalence of anemia (≤ −1.96), normal hematocrit (> −1.96 and < 1.96), and elevated hematocrit (≥ 1.96). (D) Z‐scores derived from the girls' percentile chart with two horizontal lines (−1.96 and 1.96) indicating the prevalence of anemia (≤ −1.96), normal hematocrit (> −1.96 and < 1.96), and elevated hematocrit (≥ 1.96). (E) Annual rate of change for the 50th percentile of the percentile chart for boys. (F) Annual rate of change for the 50th percentile of the percentile chart for girls.

Figure 1A,B shows the percentile charts of hematocrits of schoolchildren aged 6–18 years, and Tables 2 and 3 show the reference values by age and sex of hematocrit in each percentile extracted. The percentile charts obtained the best parameter adjustment determined by the BIC as: boys: L = 1, M = 5, S = 2; and girls: L = 1, M = 3, S = 2. Different patterns of hematocrit evolution are observed between boys and girls. Boys experience accelerated growth starting at the age of 10, which is not observed in girls. Furthermore, the curve for girls between the ages of 8 and 16 exhibits almost constant growth (with a similar derivative), in contrast to boys, who show a significant increase in growth between the ages of 10 and 14. After this period, the annual increase in hematocrit begins to slow down. Table 4 presents the differences in age percentiles between boys and girls, calculated by subtracting the age percentiles of boys from those of girls, to analyze the magnitude of sex differences. The 3rd percentile showed the greatest magnitude of differences between boys and girls. This difference varied from 3.34 (at 6 years old) to 9.77 (at 18 years old), with an increase year after year. For all other percentiles, the difference was negative between ages 6 and 11. From age 11 onwards, the difference was positive for all subsequent ages and percentiles. Figure 1C,D shows the distribution of z‐scores extracted from the percentile charts by age. The horizontal lines demarcate the z‐scores of 1.96 and − 1.96. Consequently, 58 (2.86%) boys were found to be anemic, 1955 (94.81%) had normal hematocrit levels, and 48 (2.33%) had high hematocrit levels. Girls exhibited a prevalence pattern similar to that of boys, with 73 (2.73%) anemic girls, 2534 (94.90%) with normal hematocrit, and 63 (2.36%) with high hematocrit.

**TABLE 2:** Parameters L, M, and S of the boys' percentile chart and the derived percentile values by age.

Age	L	M	S	3rd	10th	25th	50th	85th	90th	97th
6.00	0.97	36.30	0.05	32.82	33.98	35.14	36.30	37.46	38.63	39.79
7.00	0.97	36.76	0.05	33.16	34.36	35.56	36.76	37.96	39.17	40.37
8.00	0.97	37.21	0.05	33.48	34.72	35.96	37.21	38.46	39.71	40.96
9.00	0.97	37.55	0.05	33.69	34.98	36.26	37.55	38.84	40.13	41.42
10.00	0.97	37.84	0.05	33.86	35.19	36.51	37.84	39.16	40.49	41.82
11.00	0.97	38.24	0.05	34.13	35.50	36.87	38.24	39.61	40.98	42.35
12.00	0.97	38.89	0.05	34.63	36.05	37.47	38.89	40.32	41.75	43.17
13.00	0.97	39.83	0.06	35.37	36.86	38.34	39.83	41.32	42.82	44.31
14.00	0.97	40.96	0.06	36.28	37.83	39.40	40.96	42.53	44.09	45.66
15.00	0.97	42.05	0.06	37.15	38.78	40.42	42.05	43.69	45.34	46.98
16.00	0.97	42.98	0.06	37.86	39.56	41.27	42.98	44.69	46.40	48.11
17.00	0.97	43.71	0.06	38.40	40.17	41.94	43.71	45.48	47.26	49.04
18.00	0.97	44.33	0.06	38.85	40.67	42.50	44.33	46.16	48.00	49.83

*Note:* Median (M, μ), coefficient of variation (S, σ), and skewness (L, λ), the latter expressed as a Box‐Cox power.

**TABLE 3:** Parameters L, M, and S of the girls' percentile chart and the derived percentile values by age.

Age	L	M	S	3rd	10th	25th	50th	85th	90th	97th
6	1.00	36.75	0.05	32.96	34.22	35.49	36.75	38.02	39.28	40.55
7	1.00	37.13	0.05	33.23	34.53	35.83	37.13	38.42	39.72	41.02
8	1.00	37.50	0.05	33.50	34.83	36.16	37.50	38.83	40.16	41.49
9	1.00	37.85	0.05	33.75	35.12	36.48	37.85	39.21	40.58	41.94
10	1.00	38.17	0.05	33.98	35.38	36.77	38.17	39.57	40.96	42.36
11	1.00	38.46	0.06	34.17	35.60	37.03	38.46	39.89	41.31	42.74
12	1.00	38.70	0.06	34.32	35.78	37.24	38.70	40.16	41.62	43.08
13	1.00	38.89	0.06	34.43	35.92	37.40	38.89	40.38	41.87	43.35
14	1.00	39.04	0.06	34.50	36.01	37.53	39.04	40.56	42.07	43.59
15	1.00	39.16	0.06	34.54	36.08	37.62	39.16	40.70	42.24	43.78
16	1.00	39.25	0.06	34.55	36.12	37.69	39.25	40.82	42.38	43.95
17	1.00	39.33	0.06	34.56	36.15	37.74	39.33	40.92	42.51	44.10
18	1.00	39.40	0.06	34.56	36.17	37.79	39.40	41.02	42.63	44.25

*Note:* Median (M, μ), coefficient of variation (S, σ), and skewness (L, λ), the latter expressed as a Box‐Cox power.

**TABLE 4:** Variation in percentile values by age between boys and girls.

Age	3rd	10th	25th	50th	85th	90th	97th
6	3.34	−0.24	−0.35	−0.45	−0.56	−0.65	−0.76
7	3.53	−0.17	−0.27	−0.37	−0.46	−0.55	−0.65
8	3.71	−0.11	−0.2	−0.29	−0.37	−0.45	−0.53
9	3.8	−0.14	−0.22	−0.3	−0.37	−0.45	−0.52
10	3.86	−0.19	−0.26	−0.33	−0.41	−0.47	−0.54
11	4.07	−0.1	−0.16	−0.22	−0.28	−0.33	−0.39
12	4.57	0.27	0.23	0.19	0.16	0.13	0.09
13	5.4	0.94	0.94	0.94	0.94	0.95	0.96
14	6.46	1.82	1.87	1.92	1.97	2.02	2.07
15	7.51	2.7	2.8	2.89	2.99	3.1	3.2
16	8.43	3.44	3.58	3.73	3.87	4.02	4.16
17	9.15	4.02	4.2	4.38	4.56	4.75	4.94
18	9.77	4.5	4.71	4.93	5.14	5.37	5.58

*Note:* For each age and percentile, the values for boys were subtracted from those for girls.

### 3.3 Comparison of the Rate of Change of Hematocrit for Boys and Girls

Boys exhibit a subtle decline in hematocrit levels starting at age 6, with the most significant decrease occurring at 9.3 years, at a rate of 0.28% hematocrit per year. From age 10, the hematocrit level begins to rise rapidly, peaking at 13.7 years at a rate of 1.15% per year. After this peak, the rate of increase slows, stabilizing at 0.65% per year from age 17 onward. Girls exhibit a different pattern of hematocrit variation compared to boys. Their rate of change decreases from age 6 (the age of highest rate at 0.37% hematocrit per year) until it stabilizes at 0.07% per year from age 17 onward.

### 3.4 Prevalence of Anemia According to Family Economic Status

Table 5 presents the frequencies and standardized residuals of anemic and non‐anemic students (normal + high hematocrit) across different classes of family socioeconomic status. For girls, a higher prevalence of non‐anemic hematocrit was observed in class A (39.33%) compared to anemic children (23.28%), with significant standardized residuals. No significant residuals were found for the other classes. For boys, significant residuals were observed for a higher prevalence of anemic children in the lower socioeconomic class DE (13.79%) compared to non‐anemic children (5.18%), and a higher prevalence of non‐anemic children in the upper socioeconomic class A (42.63%) compared to anemic children (22.41%).

**TABLE 5:** Frequency analysis of non‐anemic and anemic schoolchildren by economic status class.

	Girls		Boys	
	Non‐anemic	Anemic	Non‐anemic	Anemic
A + B	1014 (39.33%)	17 (23.29%)	848 (42.63%)	13 (22.41%)
SR	2.77	−2.77	3.08	−3.08
C	1404 (54.46%)	48 (65.75%)	1038 (52.19%)	37 (63.79%)
SR	−1.91	1.91	−1.75	1.75
D + E	160 (6.21%)	8 (10.96%)	103 (5.18%)	8 (13.79%)
SR	−1.64	1.64	−2.86	2.86
Total	2651 (100%)	73 (100%)	1989 (100%)	58 (100%)

*Note:* Socioeconomic status classes according to the Brazilian Association of Research Companies (ABEP), being A + B (high), C (middle), and D + E (low) income classes. SR: standardized residuals. SR > |1.96| obtained by the chi‐square test were considered significant.

## 4 Discussion

Anemia, particularly in children and adolescents, remains a significant public health issue in many developing countries, with important implications for growth, cognitive development, and academic performance (Rocha et al. 2020). The percentile charts and hematocrit data presented in this study offer a detailed analysis of the developmental differences between boys and girls aged 6 to 18 years. The parameter adjustment, based on the BIC, reveals distinct growth trajectories for both sexes, with optimal parameters being L = 1, M = 5, S = 2 for boys and L = 1, M = 3, S = 2 for girls.

When analyzing the hematocrit percentile charts and reference values by age and sex, different patterns in the hematological development of schoolchildren aged 6 to 18 years are revealed. The adjustment of the LMS parameters indicates subtle but potentially significant differences in the distribution of hematocrit values between the sexes, with a slightly higher central tendency for boys. This finding aligns with the established knowledge that boys generally have higher hematocrit levels than girls, especially after puberty (Bohn et al. 2023; Rocha et al. 2020). This increase is significant compared to the levels found in girls, where the influence of testosterone is very limited. Testosterone acts as a stimulating factor for erythropoiesis, the process of red blood cell production in the bone marrow (Bachman et al. 2014). This is partly because the hormone promotes the synthesis of erythropoietin, a substance essential for the differentiation and multiplication of red blood cell precursors. The increase in hematocrit enhances oxygen transport capacity, which can be beneficial for physical performance (Al‐Sharefi et al. 2019). The greater muscle mass in boys requires more efficient oxygen transport to meet the increased metabolic demands. This leads to higher hematocrit values, reflecting a larger proportion of red blood cells in the blood, which improves oxygen delivery to the tissues (Mairbäurl 2013).

The analysis of percentiles, particularly the 3rd percentile, highlights a significant disparity between the sexes, which becomes increasingly pronounced with age. The magnitude of this difference, increasing from 3.34% at age 6 to 9.77% at age 18, suggests that boys and girls follow different hematocrit trajectories. In addition to the disparity caused by natural differences in pubertal development between the sexes, with girls generally beginning puberty earlier than boys, this variation in pubertal development may have significant implications for the interpretation of hematocrit levels and the prevalence of anemia (Chalise et al. 2018). In girls, blood loss during menstruation can significantly affect hematocrit levels. Monthly blood loss, especially in cases of heavier flows, may result in a decrease in the number of red blood cells, leading to reduced hematocrit levels (Munro et al. 2023). This effect is more pronounced if iron stores are already low, as iron is crucial for the formation of hemoglobin in red blood cells (Deloughery et al. 2023). Inadequate iron replenishment during frequent menstruation can cause anemia, characterized by hematocrit values below normal (Ekroos et al. 2024). Through Table 4, a shift is observed at age 11, where the difference between the sexes reverses from negative to positive across all percentiles, coinciding with the onset of puberty. The negative differences observed in early childhood (ages 6 to 11) may indicate that girls experience earlier or more rapid growth in hematocrit levels, possibly due to the earlier onset of puberty, which may be related to hormonal factors, growth patterns, body composition, and differing lifestyle habits between the sexes (Rosa et al. 2024).

The analysis of the z‐score, as outlined in Figure 1C,D, provides further evidence of this divergence. Z‐scores are critical for identifying outliers and assessing the distribution of hematocrit values. The proximity to normal hematocrit levels for the majority of boys (94.81%) and girls (94.90%) indicates a generally healthy population. However, the comparable prevalence of anemia (2.86% in boys and 2.73% in girls) and elevated hematocrit levels (2.33% in boys and 2.36% in girls) suggests that while most individuals fall within normal ranges, there is still a notable portion of the population that may require clinical attention.

The data reveal a higher prevalence of non‐anemic hematocrit levels among girls in socioeconomic class A + B, suggesting that children from wealthier families may have better access to nutrition, healthcare, and living conditions conducive to optimal hematological health. The relationship between anemia and socioeconomic factors is particularly relevant when considering the impacts on physical growth and cognitive development. In southern Brazil, studies such as those by (Silva et al. 2021) identify that social vulnerability in municipalities with lower economic development is associated with lower hemoglobin levels among children, highlighting the need for public policies that address both regional differences and urban–rural disparities.

For boys, the data also show a clear socioeconomic gradient, with a higher prevalence of anemia observed in the lower socioeconomic class D + E. This trend is consistent with the notion that children in lower socioeconomic classes may be more susceptible to nutritional deficiencies, infections, and other factors that can contribute to anemia. The study by (Rodrigues et al. 2011) indicates that children attending public daycare centers in the western region of Paraná (Brazil) have a high prevalence of anemia, reflecting the connection between low socioeconomic status and nutritional deficits. This finding is supported by international research, such as that by (Andriastuti et al. 2020), which demonstrates a correlation between low socioeconomic status and hematocrit levels, highlighting the critical role of socioeconomic factors in child health.

A review by (Wiafe et al. 2023) identified iron deficiency anemia (IDA) as a condition influenced by multiple factors, including dietary habits, parasitic infections, worm infestations, blood loss, and broader determinants such as educational level, socioeconomic status, rural residency, family size, and walking barefoot. Studies assessing the relationship between socioeconomic status (SES) and anemia consistently demonstrated that adolescents from lower SES backgrounds face significantly higher odds of developing anemia, with odds ratios ranging from OR = 2.16 (95% CI: 1.17–4.33) to OR = 2.86 (95% CI: 1.16–7.04) (Agrawal et al. 2018; Fentie et al. 2020). Similarly, low maternal educational attainment has been associated with a higher prevalence of anemia in adolescents. Limited formal education among mothers may hinder their ability to comprehend nutritional information and make informed dietary choices, thereby impacting household nutrition (Agrawal et al. 2018). This effect is particularly pronounced in families where mothers play a central role in food preparation and decision‐making, as maternal education has been shown to significantly influence children's hemoglobin levels. Furthermore, lower SES, often characterized by unemployment or poorly paid employment, contributes to food insecurity, inadequate dietary diversity, and insufficient nutrient intake, all of which exacerbate the risk of anemia in adolescents (Wiafe et al. 2023). A study conducted by (Soekarjo et al. 2001) in Indonesia in adolescents 12–15 years of age found that girls had a lower chance of being anemic when they had a higher socioeconomic status, and boys had a lower chance of being anemic when they had a higher socioeconomic status. (Soekarjo et al. 2001) associates these results because lower socio‐economic status reflects a lower intake of iron‐rich foods, especially sources of haem‐iron, and higher infection rates.

Menstruation has also been identified as a significant risk factor for anemia in female adolescents. Studies suggest that excessive menstrual bleeding, particularly durations exceeding five days, markedly increases the likelihood of anemia, with adjusted odds ratios (AOR) as high as 2.25 (95% CI: 1.17–4.33) (Fentie et al. 2020). These findings align with previous observations of hematocrit changes during puberty in girls, suggesting that physiological and developmental factors intersect with social determinants to influence anemia prevalence. Lower SES further compounds these risks, as it often correlates with reduced access to iron‐rich foods, particularly heme‐iron sources, and increased susceptibility.

The methodology for constructing growth centile references for individuals within a population predominantly involves two distinct approaches: the non‐parametric quantile regression method and the parametric LMS method and Generalized Additive Models for Location, Scale and Shape (GAMLSS) (Maguire 2016). Unlike the non‐parametric quantile regression method, parametric techniques present the advantage of generating a full conditional likelihood function, facilitating the creation of centiles at any desired value and enabling the likelihood estimation for any observed data point. Both the LMS and GAMLSS methods transform the data to conform to a known distribution, with the distribution parameters varying smoothly in relation to covariates (Maguire 2016). The LMS method is particularly suited for growth data characterized by skewness, assuming that the data can be normalized through a Box‐Cox power transformation. It represents a special case of the GAMLSS approach, utilizing the Box‐Cox Cole and Green (BCCG) distribution (Cole 2021). By deriving smoothed percentile curves across ages, it becomes more nuanced to identify cases of anemia that would otherwise remain undetected if fixed cutoff points for hematocrit were applied within specific age intervals. As evidenced by the percentile curves in this study, hematocrit values exhibit significant variations from year to year, spanning from childhood to adolescence, with substantial fluctuations observed during puberty. This fact reinforces the idea of detecting anemia by smooth percentile curves rather than fixed cutoff points for narrow age intervals.

The article has significant limitations regarding the etiology of anemia. The iron profile of the schoolchildren was not obtained, and other hematological parameters were not analyzed concurrently. Therefore, our statistical analysis considered only hematocrit levels below the z‐score of −1.96 as indicative of anemia, without accounting for other hematological and nutritional parameters. Defining anemia based solely on hematocrit levels may lead to underestimation of the true prevalence of anemia. The study may also underestimate the prevalence of anemia, as the percentile charts were not constructed exclusively with healthy, non‐anemic subjects. Instead, all individuals in the sample who met the inclusion criteria were included in the curve construction, rather than solely non‐anemic students. Consequently, obtaining a prevalence estimate closer to the true value would require validation with a second external sample, which was not conducted. Furthermore, some references recommend using the 5th percentile, rather than the 2.5th percentile, to screen for cases of mild anemia (World Health Organization 2024). However, due to the lack of access to clinical information and other hematological parameters of the students, the 2.5th percentile was chosen to minimize false positives and enhance the accuracy of the analysis by economic class. Future studies should include a more comprehensive assessment of nutritional and hematological status. Furthermore, hematocrit does not demonstrate superiority over hemoglobin in defining anemia (Pfeiffer and Looker 2017). Similar to the widely used HemoCue, a point‐of‐care (POC) device for quantifying hemoglobin, the i‐STAT CHEM8 + is another POC device capable of determining hematocrit within approximately two minutes (Abbot Point of Care Inc 2020). Additionally, hematocrit can be obtained efficiently and rapidly using a microhematocrit centrifuge. Consequently, both hemoglobin and hematocrit are viable and accessible parameters for defining anemia, with neither showing a clear, decisive advantage over the other. In this study, hematocrit was selected as the primary variable due to its greater availability, as hemoglobin data was partially missing from our database. Future studies could also explore the creation of hemoglobin percentile curves and compare them with our results.

Santa Cruz do Sul, a municipality located in southern Brazil where this research was conducted, has a resident population of 133 230 (IBGE, 2022), a population density of 181.54 inhabitants per square kilometer (IBGE, 2022), a school enrollment rate of 98.3% (IBGE, 2010), and a municipal Human Development Index (HDI) of 0.773 (IBGE, 2010). This information can be found on the official IBGE website, as referenced (IBGE 2025). These indicators reflect a high level of socio‐economic development and educational attainment, which likely contribute to the reduced prevalence of anemia observed. Additionally, Brazil implements the National School Feeding Program (PNAE), a cornerstone policy for ensuring food and nutrition security across the country (Ministério da Educação 2025). This program emphasizes the provision of healthy and adequate nutrition through diverse, safe, and culturally appropriate food options that respect local traditions and promote healthy eating habits. Moreover, the Brazilian Society of Pediatrics (SBP) recommends prophylactic drug supplementation for exclusively breastfed infants without risk factors, starting at 180 days of age and continuing until the end of the second year of life (Departamento Científico de Nutrologia e Hematologia 2021). For infants with identified risk factors, supplementation should commence at 90 days, regardless of dietary patterns. In alignment with these guidelines, the Brazilian Ministry of Health (MS) established the National Iron Supplementation Program in 2005, a universal and decentralized initiative aimed at preventing anemia, particularly in vulnerable groups (Ministério Da Saúde 2013). The interplay of these initiatives, alongside the methodological considerations discussed earlier, likely contributes to the low prevalence of anemia identified in this study.

This study, to the best of the authors' knowledge, represents the largest sample size to date investigating the relationship between anemia and socioeconomic status in Brazil. Moreover, it marks the first instance in which hematocrit variation rate curves have been generated for schoolchildren in Brazil, enabling their analysis across different pubertal stages. The resulting percentile curves facilitate the longitudinal monitoring of schoolchildren and serve as a potential tool for anemia screening.

## 5 Conclusions

The percentile charts fitted by the LMS method obtained a satisfactory percentile adjustment to determine the prevalence of anemia. In the total sample, 2.86% and 2.73% of anemia were verified for boys and girls, respectively. A higher prevalence of anemia was found among boys in lower socioeconomic classes, while girls in higher socioeconomic classes showed a lower prevalence of anemia. Furthermore, it was found that boys experience a slight decline in hematocrit levels starting at age 6, with the sharpest drop at 9.3 years. From age 10, their hematocrit levels rise rapidly, peaking at 13.7 years before stabilizing at a slower increase from age 17. In contrast, girls show a different pattern, with a gradual decrease in the rate of change from age 6, stabilizing at age 17. Hematocrit percentile charts are therefore clinically useful for screening anemia in children and adolescents, as hematocrit is a quick and low‐cost test.

## Author Contributions

**Vanessa Regina Jung:** conceptualization, investigation, and writing – original draft. **Nikolas Mateus Pereira de Souza:** conceptualization, methodology, formal analysis, and writing – original draft. **Dhuli Kimberli Abeg da Rosa:** formal analysis and data curation. **João Francisco de Castro Silveira:** writing – review and editing. **Cézane Priscila Reuter:** writing – review and editing and supervision. **Alexandre Rieger:** formal analysis, writing – review and editing, project administration, and supervision.

## Conflicts of Interest

The authors declare no conflicts of interest.

## Supporting information


Data S1.

## Data Availability

The data that support the findings of this study are available from the corresponding author upon reasonable request.
