# Supplementary material for: Detection of Anemia in Schoolchildren Aged 6–18 Years With Hematocrit Percentile Charts and the Impact of Economic Status in Southern Brazil
Source: Am J Hum Biol. 2025 Mar 31;37(4):e70034. doi: 10.1002/ajhb.70034 (PMC11959109; doi:10.1002/ajhb.70034)
Supplement: Supplementary file 1 — Data S1. [file AJHB-37-e70034-s001.docx]

**Table S1** The simplified classification of economic status proposed by the Brazilian Association of Research Companies (ABEP) between 2014-2017

| **Socioeconomic status** | **Average family income (R$)** | **Correspondence in dólar (US$)** |
| --- | --- | --- |
| **A** | 20,888-23,345 | 9,366- 7,530 |
| **B1** | 9,242-10,386 | 4,144- 3,350 |
| **B2** | 4,852-5,363 | 2,175- 1,730 |
| **C1** | 2,705-2,965 | 1,213- 956 |
| **C2** | 1,625-1691 | 728-545 |
| **D-E** | 768-708 | 344-288 |

For comparison purposes, the corresponding value of the dollar in june 2014 and june 2016 was considered.
